# The District Nurse and the Measles Campaign

**Published:** 1920-01-17

**Authors:** Amy Hughes, Zoe L. Puxley

**Affiliations:** *Joint Hon. Secretarie.* Central Council for District Nursing in London.; *Joint Hon. Secretarie.* Central Council for District Nursing in London.


### THE EDITOR'S LETTER-BOX.

**[Correspondence on all subjects is invited, but we cannot in any way be responsible for the opinions expressed by our correspondents, who must give their name and address as a guarantee of good faith, but not necessarily for publication. Correspondents are reminded that brevity of style and conciseness of statement greatly facilitate early insertion.]**

### THE DISTRICT NURSE AND THE MEASLES CAMPAIGN.

*To the Editor of* The Hospital.

Sir,—The attention of the Central Council for District Nursing in London has been called to your article on "The Campaign against Measles" in your issue of the 3rd instant. The paragraph headed "Nursing" suggests that district nurses are not allowed to attend infectious cases, which makes it impossible for the nursing of measles to be carried out by them. It would probably be of interest to you to see the enclosed report, which was brought out by the Nursing Council and circulated amongst all the District Nursing Associations of London. As a result of this, and various conferences which have been held from time to time with medical officers of health and others concerned, arrangements have now been made by the Councils of practically every borough in London for the District Nursing Associations to undertake the nursing of measles by arrangement with the local authority. The rules laid down in the report have been widely adopted, and the Council are assured that no ill results have followed the new policy, accompanied by suitable precautions. You are probably aware that all District Nursing Associations employing fully trained nurses in the County of London are represented on the Council, so that the above statement refers equally to the Ranyard Nurses, the Associations affiliated to the Queen Victoria's Jubilee Institute, and the other recognised non-affiliated Associations.—Yours faithfully,

Amy Hughes,
Zoe L. Puxley,
*Joint Hon. Secretaries.*

Central Council for District Nursing in London.
January 12, 1920.

[The author of our article suggests that some special arrangement ought universally to be made that district nurses may reasonably attend both measles and ordinary cases—when proper precautions are taken. That these arrangements should be adopted everywhere is evidenced by the experience already gained on the working of the excellent recommendations of the Central Council.—Ed. The Hospital.]

